# Morpho-histology and genotype dependence of in vitro morphogenesis in mature embryo cultures of wheat

**DOI:** 10.1007/s00709-014-0647-7

**Published:** 2014-04-25

**Authors:** Fabienne Delporte, Anna Pretova, Patrick du Jardin, Bernard Watillon

**Affiliations:** 1 Department of Life Sciences, Bioengineering Unit, Walloon Agricultural Research Centre (CRA-W), Chaussée de Charleroi 234, 5030 Gembloux, Belgium; 2 Institute of Plant Genetics and Biotechnology, Slovak Academy of Sciences, Akademicka 2, P.O. Box 39 A, 950 07 Nitra, Slovakia; 3 Gembloux Agro-Bio Tech, Plant Biology Unit, University of Liège (ULg), Passage des Déportés, 2, 5030 Gembloux, Belgium; 4 Department of Biology- Faculty of Natural Sciences, University of SS Cyril and Methodius in Trnava, Nám. J. Herdu 2, SK 917 01 Trnava, Slovak Republic

**Keywords:** Tissue culture competency, Differentiation, Ontogenesis, Regeneration, Somatic embryogenesis, Totipotency

## Abstract

Cellular totipotency is one of the basic principles of plant biotechnology. Currently, the success of the procedure used to produce transgenic plants is directly proportional to the successful insertion of foreign DNA into the genome of suitable target tissue/cells that are able to regenerate plants. The mature embryo (ME) is increasingly recognized as a valuable explant for developing regenerable cell lines in wheat biotechnology. We have previously developed a regeneration procedure based on fragmented ME in vitro culture. Before we can use this regeneration system as a model for molecular studies of the morphogenic pathway induced in vitro and investigate the functional links between regenerative capacity and transformation receptiveness, some questions need to be answered. Plant regeneration from cultured tissues is genetically controlled. Factors such as age/degree of differentiation and physiological conditions affect the response of explants to culture conditions. Plant regeneration in culture can be achieved through embryogenesis or organogenesis. In this paper, the suitability of ME tissues for tissue culture and the chronological series of morphological data observed at the macroscopic level are documented. Genetic variability at each step of the regeneration process was evaluated through a varietal comparison of several elite wheat cultivars. A detailed histological analysis of the chronological sequence of morphological events during ontogeny was conducted. Compared with cultures of immature zygotic embryos, we found that the embryogenic pathway occurs slightly earlier and is of a different origin in our model. Cytological, physiological, and some biochemical aspects of somatic embryo formation in wheat ME culture are discussed.

## Introduction

With few exceptions, plant genetic engineering requires using molecular biology tools along with the technology for gene transfer into the plant genomic DNA, combined with robust protocols allowing whole plants to be regenerated from the genetically modified cells. In vitro plant regeneration techniques and models are also widely used as large-scale propagation tools and case studies for basic research in plant development, as well as in response to environmental factors.

Plant regeneration in culture can be achieved through embryogenesis (i.e., the developmental process by which bipolar structures that are similar to zygotic embryos are developed from haploid or diploid somatic cells through the characteristic embryological stages without gamete fusion) or organogenesis (i.e., through the formation of unipolar primordia, which subsequently undergo organogenesis, resulting in adventitious shoot or root regeneration) (Jia et al. 2009; Hicks 1994).

Morphogenesis in vitro is a complex process affected by several endogenous and external factors with cumulative effects. “Expression of the embryogenic/organogenic potential only occurs if cells within the explant are ‘competent’ or responsive to specific culture cues which allow them to differentiate into embryos or organs” (Elhiti and Stasolla 2011). Factors such as age, ontogenic and physiological conditions, and the degree of differentiation affect the response of the explants to in vitro culture conditions.

For many recalcitrant species, such as cereals, cotton, and pines, immature zygotic embryos have long been considered as the most suitable explants with which to establish regenerating cultures because of their juvenile nature (Bhojwani and Razdan 1986). As a general rule, younger tissues, such as embryonic tissues in zygotic embryos, have greater potential and ability to produce embryos and organs compared with more differentiated mature tissues. In recent years, immature and mature zygotic embryos have been the preferred choice of explants for an increasing number of angiosperm and conifer species (Elhiti and Stasolla 2011; Tahir et al. 2011).

The use of mature embryos (MEs) as starting material provides a low-cost and time-saving alternative to using immature embryos (IEs) for wheat biotechnology development (Xia et al. 2012). The convenience of regenerating plantlets from the rice MEs has made this species a model organism for applied biotechnology and genetic engineering study in cereals (Yang et al. 2013; Shrawat and Good 2010; Toki et al. 2006; Xia et al. 2012; Jeong et al. 2002; Lee et al. 1999; Nakamura et al. 2007; Saika and Toki 2010; Sallaud et al. 2004). In rice, *Agrobacterium*-mediated transformation using ME-derived calli is becoming the method of choice for most laboratories.

Highly efficient rice transformation protocols using mature seed-derived calli have enabled several thousand independent lines to be generated. This striking progress in rice genetic engineering technology has led to the production of large populations of transgenic plants, which are needed especially for large-scale applications such as high-throughput functional analysis, T-DNA insertion mutagenesis, gene targeting, and genome editing (Nakamura et al. 2007; Saika et al. 2011; Saika and Toki 2009, 2010; Sallaud et al. 2004; Terada et al. 2004; Yang et al. 2004).

In wheat, zygotic IEs (and derived calli), considered to be the most responsive explants, have been the usual target tissues for genetic transformation through microprojectile or *Agrobacterium*-mediated DNA delivery (Shewry and Jones 2005; Vasil 2005; Xia et al. 2012). MEs are now increasingly recognized as valuable explants for developing regenerable cell lines in wheat biotechnology. This is illustrated by the numerous studies conducted in recent years on optimizing in vitro culture conditions for callus induction, plant regeneration, and transformation parameters of ME-based cultures. Among the many processes and factors being evaluated in order to optimize the overall regeneration capacity of elite local wheat cultivars worldwide are genotype screening and genetic influence, inoculation method and explant treatment, preculture incubation time and conditions, different media with organic/inorganic additives, and varying phytohormone concentrations and combinations throughout successive steps of the morphogenic process (Battal 2010; Coskun et al. 2013; Ding et al. 2009; Miroshnichenko et al. 2009, 2011, 2013; Moghaieb et al. 2010; Murín et al. 2012; Ozbay and Özgen 2010; Parmar et al. 2012; Rashid et al. 2009; Ren et al. 2010; Tang et al. 2006; Yin et al. 2011; Yu et al. 2008).

We have previously developed a simple and alternative procedure for wheat, based on MEs (Delporte et al. 2001). Our results showed that cell lines that were induced in vitro by 2,4-dichlorophenoxyacetic acid (2,4-D) presented variable ability in gene transfer over the successive steps of the regenerative pathway. In a comparative study, we also observed varying abilities in transient and stable marker gene expression between callus cells derived from zygotic IEs and those derived from zygotic MEs, with a lower proportion of cell lines produced from MEs expressing a marker gene, but those expressing calli displaying a greater number of stable GUS-expressing cells (Delporte et al. 2005).

In order to investigate the fundamentals and the molecular events underlying the morphogenic pathway induced in vitro, and the effect of the developmental route on competence in gene transfer and stable transformation (Delporte et al. 2013), a more comprehensive study of the regeneration process was needed.

The acquisition of regeneration potential is determined at several levels, with genotype being the most important factor. This is easily demonstrated by the variation in response often observed among different genotypes within the same species and its heritability. It has also been confirmed by the development of molecular tools (e.g., quantitative trait loci [QTL] mapping technology), especially in monocots (reviewed by Bolibok and Rakoczy-Trojanowska (2006)). Extensive research has been conducted to identify the genetic mechanisms underlying wheat tissue culture response (TCR) and the QTLs associated with both immature and mature embryo TCR. Jia’s group studies highlighted the existence of wide genetic variations in varieties and showed that these variations are linked to multiple chromosome regions, TCR being under polygenic control (Jia et al. 2007, 2009). At present, however, it is impossible to predict the exact growth medium and protocol for callusing and regeneration from a particular explant of specific wheat cultivars.

There can be large genotype-dependent variations in wheat TCR. These variations hamper wheat genetic improvement programs that depend greatly on tissue culture procedures. From a biotechnology perspective, we are interested in working with local germplasm that has not yet been considered in transformation experiments.

In this study, commercial wheat varieties adapted to local European environments were chosen randomly, and their responses to in vitro culture conditions were compared. Their potential and ability to produce regenerable tissues throughout the process, and ultimately their overall regeneration competence, were measured. This information is crucial for selecting promising genotypes suitable for genetic transformation, and subsequently for designing optimized protocols in a genotype-dependent way, as well as in order to target the best physiological state for performing gene transfer and to better understand and meet the specific requirements of each developmental transition stage. The process of making a transgenic plant can stall at several key points, starting with the acquisition of a competent totipotent state (the acquisition of a stem-cell-like state in cells as they reenter the cell cycle), the progression of cells through the S phase of the cycle (which makes cells suitable for transformation), and thereafter the differentiation of the transformed cells into new meristems or embryos (Arias et al. 2006).

Basic questions also remained about why cells from a zygotic embryo of the same organism (same species, genus, and variety) behaved differently during transformation (Delporte et al. 2005) (i.e., whether discrepancies depended on the developmental history at the time of sampling—physiological state, mature vs. immature—or whether different developmental pathways, origins, and chronology were involved).

In this study, the chronological sequence of morphological events was observed at the macroscopic level, and a detailed histological analysis of the ontogeny was conducted (in order to determine the nature of the morphogenic response—organogenic or embryogenic—and to identify the tissue/cellular types involved in plant neoformation), when ME tissues were induced in culture.

The paper concludes with a discussion of cytological, physiological, and some biochemical aspects of somatic embryo formation in wheat ME culture.

## Material and methods

### Plant material

Commercial wheat seeds (*Triticum aestivum* L.) of one spring and three winter cultivars (Minaret, Dream, Derwent, and Petrus, respectively) were supplied by the plant breeding companies Clovis Matton (Avelgem–Kerkhove, Belgium) and Saatzucht Schweiger GBR (Moosburg, Germany). The donor plants were grown in the field. Harvested mature seeds were stored at room temperature.

### Seed surface sterilization and aseptic embryo isolation

Wheat caryopses were fungicide treated (Sibutol® Bayer) against seed-borne pathogenic fungi at least 8 days before culture initiation, after which the seeds were kept in a flask for several weeks for subsequent use. After a 16-h rehydration in sterile water at room temperature, the seeds were surface-sterilized with 70 % ethanol for 2 min, soaked in 8 % calcium hypochlorite containing 0.1 % Tween 80 (Merck) for 10 min, and rinsed three times with deionized sterile water. The embryos were aseptically removed and protected from desiccation.

### Tissue culture

For culture initiation, 100 embryos were ground through a sterile nylon mesh (approximately 600 μm porosity). The resulting embryo fragments (500 μm mean diameter) were washed twice with 20 ml of a liquid basal medium (i.e., MS medium, supplemented with 100 mg/l casein hydrolysate [Sigma] and 20 g/l sucrose [Merck] as the carbon source, pH adjusted to 5.8 with NaOH prior to autoclaving). The callus induction medium was the liquid basal medium containing 2 mg/l filter-sterilized 2,4-D (Sigma) as the growth regulator, semi-solidified with 0.7 % agar (Invitrogen). Thin tissue fragments (≈500 fragments resulting from crushing 100 embryos) were resuspended in 4 ml of the liquid basal medium. The resulting suspension was distributed among five 9-cm disposable plastic Petri dishes containing the callus induction medium. Excess liquid medium was retrieved and discarded before sealing the dishes. Light and temperature conditions for callus culture and plant regeneration were as described earlier (Delporte et al. 2005). Plant growth and rooting were achieved in tubes containing one-half-strength MS medium.

### Varietal study

Tissue fragments from 20 embryos were plated on culture media and grown in a randomized complete block with seven replications. The variables for this experiment included the rate of callus induction at 1 week of culture, the percentage of embryogenic callus at 4 and 7 weeks, and the frequency of plant regeneration.

### Histological study

The regeneration process was studied by histological observations, using light microscopy (Nikon Eclipse E 400 microscope), of proliferating tissues (*T. aestivum* L. Dream cv) sampled at regular intervals.

The identification of the morphogenic pathway and the localization of its initiation were considered jointly. For this purpose, the samples were derived from MEs kept whole or dissected. In the latter case, zygotic MEs were dissected under a binocular microscope in order to separate the main constituent tissues (scutellum, as well as epicotyl, hypocotyl, or mesocotyl attached or not to pieces of scutellum) that were cultured separately.

The specimens were bathed and fixed in a formalin-acetic acid-alcohol (FAA) solution (5 % formol, 5 % acetic acid, 90 % of 70 % ethyl alcohol; *v*/*v*) for at least 24 h at 4 °C, rinsed, then dehydrated through a graded ethanol series (butanol/ethyl alcohol series), and finally infiltrated with and embedded in a paraffin matrix (57/60 °C melting temperature, Merck). Sections (12 μm) were taken using a rotary microtome (Microm) and were mounted on glass slides. Structural elements were stained with a fixative acid alcoholic solution of 1 % orcein. Images were captured using a reflex digital camera (Nikon Coolpix 4500).

### Statistical analysis

Calculated variables were normalized (arcsinrac[x] variable transformation), and the data were analyzed by analysis of variance. Mean differences were compared pairwise using the Tukey’s multiple comparison procedure (Systat for Windows, Version 8, SYSTAT Software Inc.).

## Results

### Morphological events: macroscopic observations

The tissue culture and regeneration protocol we had previously developed used mature caryopses ground to pieces as the starting material (Delporte et al. 2001).

**Fig. 1:**
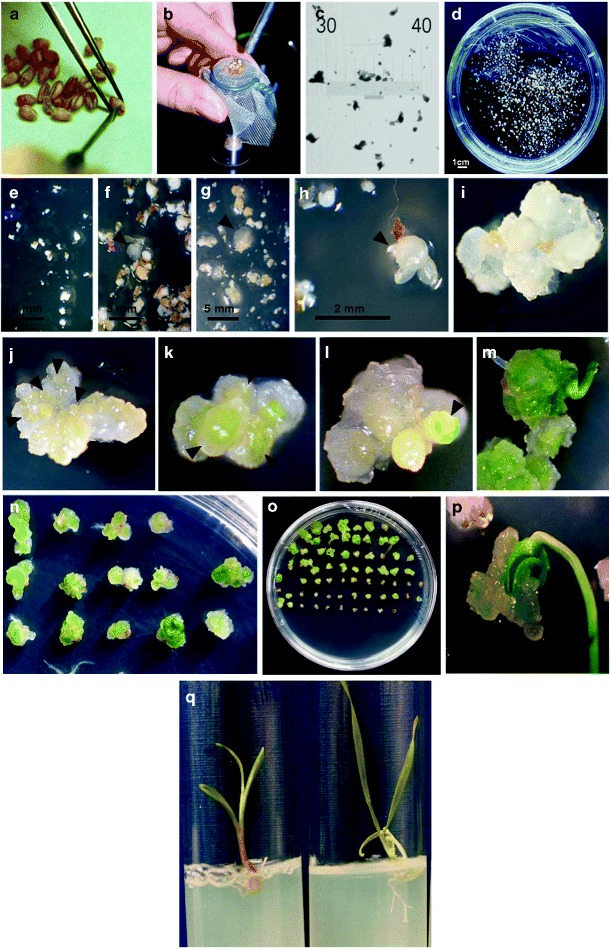
Tissue culture based on fragmented mature embryos: methodology, morphogenic pathway of the initiated calli, and shoot regeneration. Aseptic embryo isolation with a lancet under a stereo microscope (**a**), grinding by extrusion through a sterile nylon mesh, using the rounded tip of the handle of a lancet (**b**), the resulting mature caryopses reduced to fragments: tissue fragments laid down on micrometric cover glass (**c**) and in suspension in the liquid basal medium (in a 9-cm Petri dish) before induction in in vitro culture (**d**). Scheduling of tissue culture within the time scale of 1 week (**e**–**h**) and 2–8 weeks (**i**–**p**). 2,4-D cell proliferation induction in explants after 1, 4, and 7 days of culture in darkness, respectively (**e**–**g/h**). Numerous actively proliferating specimens visible after 4 days (**f**) doubled their volume within 3 days (**g**); some of these 7-day-old structures, densely formed with actively dividing cells, appeared as compact and were considered morphogenic (**h**). Nodular appearance calli visible 2 weeks after the 2,4-D induction (**i**). Clusters of yellowish-white nodular structures (**j**), chlorophyll synthesis (large green pigmented areas within the nodular structures, **k**), development of organs (green tissue rising up to the surface, **l**), leaf emergence and expansion (**m**–**o**), and plantlet regeneration (**p**) after the calli were transferred to a medium without growth regulators and exposed to light. Healthy plantlets and root development on the rooting medium (**q**: plantlet on the *left* starting to develop roots; plantlet on the *right* showing slightly more developed roots, with lateral growth)

Figure 1 shows the protocol scheduling, illustrates the nature of explants induced in culture, and describes the chronology of the morphogenic events observed at the macroscopic scale (Fig. 1a–q).

The 2,4-D cell division induction in explants produced calli within 1 week. Cell proliferation became visible after day 4 (Fig. 1f), and actively proliferating specimens doubled their volume within 3 days (Fig. 1g). As early as 1 week after inoculation, dense structures of increasing size were clearly visible to the naked eye and were considered organogenetic/embryogenetic (Fig. 1h), in line with commonly used descriptions.

After 2 weeks of induction, calli with a nodular appearance were formed (Fig. 1i). After their transfer to a medium without growth regulators and their exposure to light, clusters of yellowish-white nodular structures appeared (Fig. 1j), and chlorophyll synthesis became obvious (as large green pigmented areas within the nodular structures, Fig. 1k), followed by shoot emergence (green tissue rising up to the surface, Fig. 1l) and leaf expansion (Fig. 1m–o). Subsequently, the regenerated plantlets (Fig. 1p) were transferred into tubes for growth and rooting (healthy plantlets starting to develop roots, Fig. 1q).

Regeneration occurred within a short time (a few weeks), starting with green shoot emergence, followed by rooting.

Acclimated plants appeared to be normal phenotypically and were fertile: they developed normally, flowered, and set seed, and several tillers per plant produced fertile ears. The seeds were viable, able to germinate and develop into a complete plant.

### Genetic determinism of morphogenetic events: varietal comparison

Responsiveness to in vitro culture induction was tested for four genotypes of agronomical value. Wheat varieties were randomly selected and represented two groups, spring and winter commercial varieties, cultivated in European countries (Table 1).

**Table 1:** Commercially grown European wheat cultivars tested in the varietal study on tissue culture behavior and regeneration ability

Cultivar name	Country/year of origin	Type
Derwent	GBR/1978	W
Dream	DEU/1999	W
Minaret	FRA/1982	S
	NDL/1983	
Petrus	DEU/1996	W

Based on information from http://genbank.vurv.cz/wheat/pedigree/pedigree.asp and the European Wheat Database (EWDB) http://genbank.vurv.cz/ewdb/

*W* winter, *S* spring, *DEU* Germany, *FRA* France, *GBR* United Kingdom, *NDL* Netherlands

**Fig. 2:**
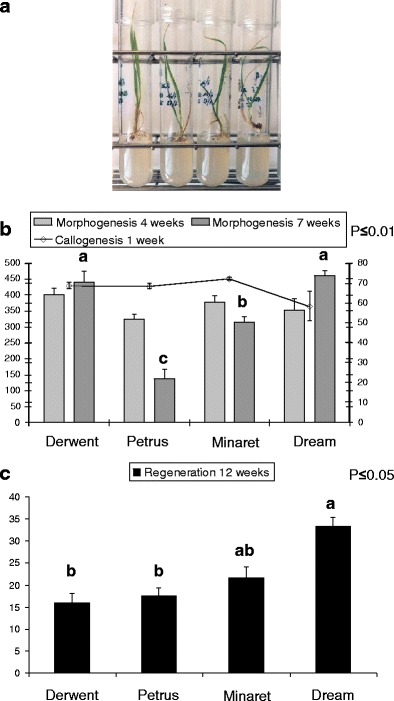
Responsiveness of commercial wheat genotypes to in vitro culture and regeneration from mature embryo tissues. Growth and rooting of completely formed plantlets (**a**), callogenesis and embryogenesis (**b**), and regeneration (**c**) frequencies. *Callogenesis* Number of actively proliferating calli/number of mature embryos × 100, 1 week after culture initiation (*line graph*, *left axis*). *Morphogenesis* Number of organogenic/embryogenic calli per number of calli induced × 100, 4 and 7 weeks after culture initiation (*bar graph*, *right axis*). *Regeneration* Number of plants regenerated/number of mature caryopses × 100, at the end of the experiment. *Bars* represent the standard deviation of the mean for seven replicates (20 embryos per replicate); *data with different letters* are significantly different at *p* ≤ 0.01 (**b**) and *p* ≤ 0.05 (**c**)

The in vitro competence of each wheat accession was assessed according to the morphogenetic behavior of the explants throughout the culture process (as illustrated in Fig. 1f–q) and their shoot production efficiency (Fig. 2).

All the genotypes showed excellent competence for “callogenesis” (undifferentiated cellular proliferation, Fig. 1f, g) and “morphogenesis” (white to greenish-white, compact and smooth, plane or dome-shaped or nodular appearance calli formed with densely proliferating cells, Fig. 1h–j). Those calli termed “organogenic/embryogenic” were prone to engage in differentiation, confirmed by cell cluster greening (i.e., chloroplast differentiation, Fig. 1k) and followed by shoot emergence (Fig. 1l–p) when exposed to regenerative conditions (transfer to medium without growth regulator and exposure to light). The calli considered “non-morphogenetic” were white, limpid, watery, friable, and formed with large highly vacuolated cells (data not shown).

Regeneration was observed (fully developed plantlets, Figs. 1q and 2a) in all the genotypes tested. Plantlet emergence could span several weeks (i.e., 6 to 12 weeks after initiation, with a production peak in the 8–10 interval).

The genotypes were all able to regenerate, but with varying potential, as shown by the varying conversion rates from callogenesis to morphogenesis and from the latter into plantlets.

First, all the genotypes responded identically (*p* ≤ 0.05) with regard to calli production, notably the organogenetic/embryogenic type, after 1 and 4 weeks of exposure to 2,4-D, respectively. On average across all the genotypes, 90 % of the tissue fragments proliferated within 1 week of induction, and a significant proportion of these calli were morphogenetic (almost 60 %) after 4 weeks of culture (Fig. 2b).

Second, after the transfer of these tissues to regenerative conditions, the varieties showed highly significant differences in organogenetic/embryogenic calli production (*p* ≤ 0.01) after 7 weeks of culture. On average across all the genotypes, ~55 % of the calli were morphogenetic, but the individual values fluctuated from 20 to 70 %. Dream and Derwent were the best suited to forming morphogenetic calli, whereas Petrus was the least suited (Fig. 2b).

Later, these distinct types of behavior were confirmed by shoot production efficiency, apart from Derwent (Fig. 2c). More explicitly, the rate of formation of morphogenetic calli in three out of four varieties (at 7 weeks of culture) reflected the subsequent regeneration efficiency. Although Derwent presented a similar morphogenetic response to Dream, its ability in terms of further differentiation and shoot regeneration was lower.

Ultimately, all the cultivars tested in this experiment produced plants, at an average rate of 22 % (i.e., their values ranged between 15 and 35 %, *p* ≤ 0.05) (Fig. 2c).

### Embryogenic differentiation: histological study

To conduct a histological characterization of the in vitro ontogenic process involved in plant neoformation, sections were made after varying culture periods in calli deriving from whole or dissected embryos, allowing the timing and tissue origin of the regeneration pathway to be investigated jointly.

Embryogenic cultures remain the most reliable and most frequently used route in cereal regeneration (Vasil 2005). Plants can regenerate, however, by somatic embryogenesis or by adventitious bud and shoot development, with subsequent rooting (Bhaskaran and Smith 1990).

Organogenesis is defined as the production of unipolar structures, the shoot or root apical meristems, which subsequently undergo organogenesis that results in adventitious shoot or root regeneration. In many instances, shoot primordia are formed first, followed by leafy vegetative shoots, which are then rooted via root organogenesis (Elhiti and Stasolla 2011). The presence of the shoot apical meristem and vascular connection with maternal explants indicate that regenerants are shoots or shoot-like structures produced through organogenesis (Salaj et al. 2005).

Somatic embryogenesis is defined as a multistep process in which a bipolar structure, resembling a zygotic embryo, develops from a non-zygotic cell (i.e., haploid or diploid somatic cells), without a vascular connection with the original tissue, through a chronological series of characteristic embryological stages without the fusion of gametes (Jiménez 2001; von Arnold et al. 2002).

**Fig. 3:**
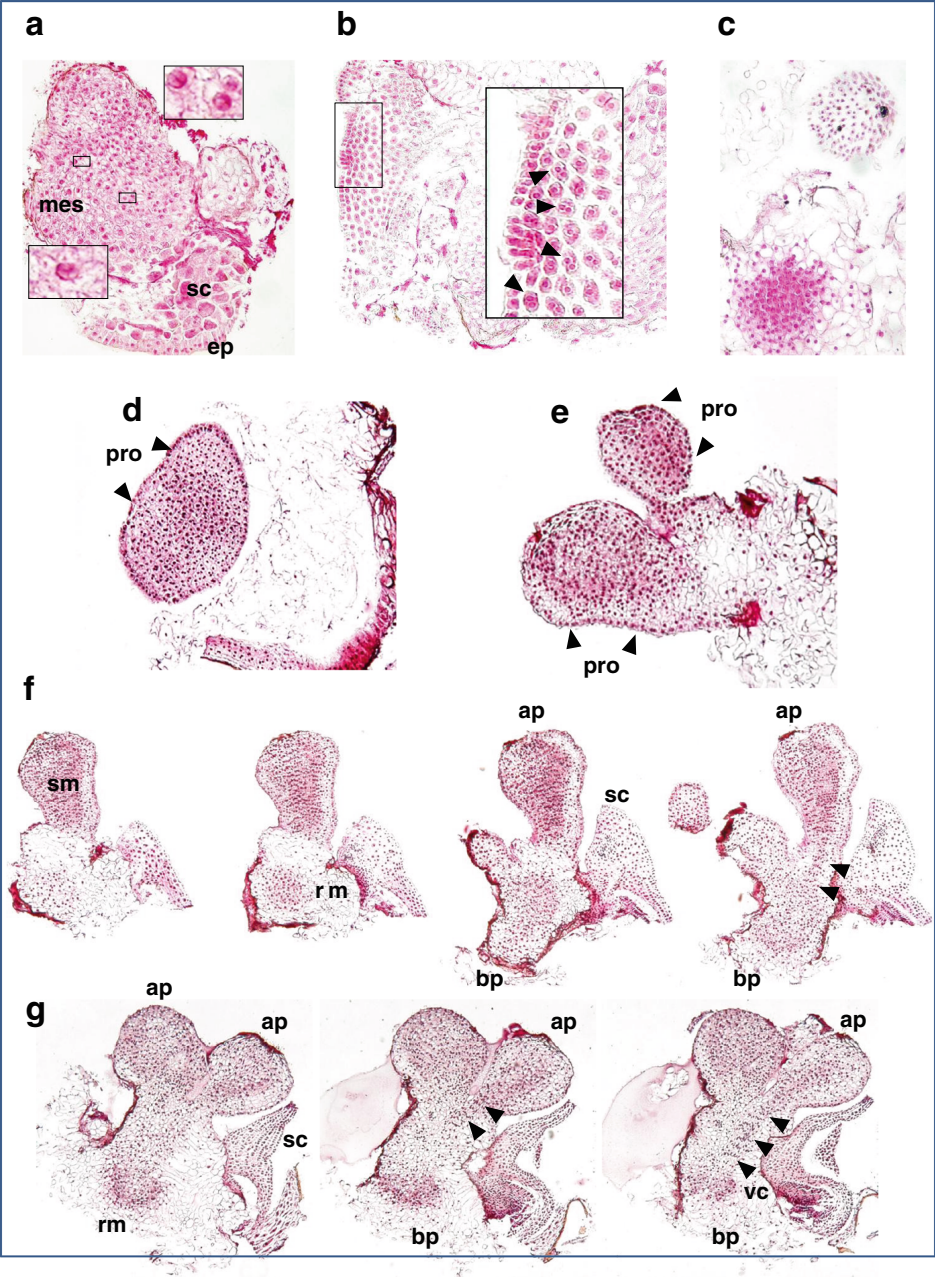
Regeneration process in wheat mature embryo tissues: somatic embryogenesis—intermediate steps of somatic embryo development. Initiation of cell division 1 DAC (**a**), small clusters of a few dividing cells. Cell proliferation, first clues apparent 2 DAC (**b**) and clumps of highly meristematic cells apparent 6 DAC (**c**). Globular embryo (pre-embryo); a widened globular structure bordered by a well-developed protoderm (the outer unicellular layer, *arrowed*), clearly delimited from the callus 8 DAC (**d**). Polar structure without vascular connection with the original tissue 12 DAC; a polarized embryonic structure next to a globular one (**e**). Bipolar structure, with root meristem differentiation at the basal pole; the organized formation next to this basal part represents the scutellum, indicating a level of further differentiation (**f**). Bipolar structures, well-organized somatic embryos; in one of the two shown, the vascular connections are visible between the apical and basal poles (*arrowed*), with a scutellum next to its basal part (**g**). Three major embryonic tissue systems—shoot apical meristem, root apical meristem, and the differentiation of procambial strands—are visible. In order to better describe post-globular development of the embryo, we have assembled a series of representative pictures of the same object, sequentially ordered, so that different planes can be observed (the different structures appear in different sections because they have not developed in the same plane). DAC days after culture initiation, *mes* mesocotyl, *sc* scutellum, *ep* scutellar epithelium of the original zygotic embryo, *pro* protoderm, *sm* shoot meristem, *rm* root meristem, *ap* apical pole, *bp* basal pole, *vc* vascular connections

**Fig. 4:**
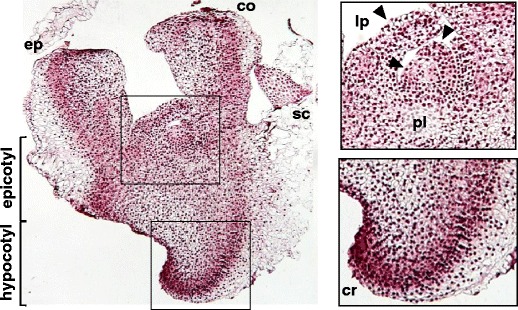
Histological observation of fully developed somatic embryo. Bipolar, mature somatic embryo in culture showing all the characteristic organs of grass embryos 21 DAC. *co* coleoptile, *ep* epiblast, *sc* scutellum, *pl* plumule, *lp* leaf primordial (*arrows*), *cr* coleorhiza

In this study, we were able to follow the sequential formation of somatic embryos from their early development (first cellular divisions) to the final step of complete differentiation (Figs. 3 and 4):

(i) Initiation of cell division: 1 day after 2,4-D induction, a few dividing cells were observed (Fig. 3a).
(ii) Cell proliferation: after 2 days of culture, the first signs of a more active cellular division were visible (Fig. 3b), sometimes producing small alignments in pairs of dividing cells, which appeared isolated from their neighboring cells; after 6 days, intensified cell divisions were visible; repeated mitotic divisions in any direction produced progressively enlarged clusters of meristematic cells (pro-embryogenic clumps) (Fig. 3c). These cell clusters might have been formed from the division of an initial single cell.
(iii) Globular embryo: at day 8, the first signs of differentiation appeared; a unicellular layer (pro: protoderm) enveloped the pro-embryo, separated and clearly distinct from the callus cells (Fig. 3d).
(iv) Polar structure: after 8 days, the process of differentiation was confirmed and was followed by polarized development; the formation of one apical meristem was clearly distinct and had no vascular connection with the original tissue (Fig. 3e).
(v) Bipolar structure: at about 12 days, an apical-basal pattern of symmetry was established and an embryo axis was formed (consisting of the shoot and root apical meristems), with pro-cambial strand initiation between the two opposite poles; in addition to this bipolarization and the establishment of future vascular tissues, a level of further differentiation was indicated by adjacent structural elements assimilated to the scutellum of the zygotic embryo (Fig. 3f, g).
(vi) Mature somatic embryo: showing all the characteristic organs of grass embryos, plumule with the coleoptile enclosing the leaf primordia, coleorhiza, scutellum, and epiblast (Fig. 4).

In addition, in order to determine the origin of cells involved in inducing somatic embryogenesis, MEs were dissected and the main constitutive tissues of their anatomical structure were used as starting material. From an examination of these specimens (hypocotyl, mesocotyl, coleoptile, and scutellum alone or hypocotyl, mesocotyl, and coleoptile separated from each other without the scutellum having been removed; data not shown), we observed that the embryogenic pattern was preferentially formed by the proliferation of the cells in the vicinity of the scutellar node (mesocotyl) and when some scutellum tissue still adhered to the explant (i.e., when this organ had not been removed before taking the mesocotyl tissue away from the embryonic axis).

The ontogeny of somatic embryos occurred within a short period, from the meristematic type proliferation (unorganized cell division, the first 6 days of culture) to the building of the root/shoot axis, which is the main achievement of plant embryogenesis (fully differentiated somatic embryos were visible after 12 days). Within this period, the demarcation of the protoderm layer marked the beginning of the structural differentiation during the globular stage (after 8 days).

## Discussion

### Mature zygotic embryo, a valuable prospect for biotechnology

Very efficient tissue culture protocols and high-throughput transformation platforms are needed for large-scale applications in plant biotechnology and high-throughput functional analysis. A highly efficient and reproducible regeneration method is still lacking for Triticeae, although such high-frequency regeneration protocols have been developed for rice, based notably on the culture of mature seeds. The ME is arguably one of the best suited explants for plant biotechnology because of its unlimited availability and lack of growth season restriction. An efficient regeneration system using the MEs of wheat (*T. aestivum* L.) is still not available.

In this study, drawing on a previously described protocol for somatic embryogenesis induction established from wheat ME culture, (i) the competence of ME tissues for tissue culture and morphogenesis was documented, (ii) genotypic variation at each step of the regeneration process was evaluated through varietal comparison, and (iii) a detailed histological study of the oncogenesis involved in plant neoformation was conducted.

### Wheat mature zygotic embryo: tissue culture competence and morphogenesis

Not all plant cells express totipotency in vitro. Whatever the developmental process, the ability to regenerate a large number of plants from in vitro cultured cells and tissues depends on the genetic background, the physiological status of the donor plant, the organ used as the explant and its physiological state/development, the culture medium and conditions, and the interactions among these factors (Vasil 2008; Finer 2010).

Somatic cells of monocotyledonous plants differentiate early and rapidly, followed by the loss of their mitotic and morphogenetic capabilities. The early loss of totipotency in monocots might be linked to the strict regulation of the synthesis and/or metabolism of endogenous growth regulators such as auxin (Fehér 2006). Since the preferential synthesis of auxin in plants occurs in young and developing tissues and organs, it is assumed that zygotic embryos have higher levels of this growth regulator compared with more mature explants (Elhiti and Stasolla 2011).

In grasses, for example, only zygotic embryos, inflorescences, and tissue derived from juvenile shoots can be used to obtain regenerable cultures. All these explants contain meristematic cells that are targets for plant growth regulator action (Bhaskaran and Smith 1990; Fehér 2006; Vasil 1988).

Although exogenous auxin is required to induce and maintain a high rate of unorganized growth in plant cell cultures, a low-auxin or hormone-free medium is required to induce developmental responses that are usually dependent on endogenous hormonal factors (Fehér 2006).

In this study, we documented and illustrated the successful establishment of morphogenic and regenerable cell cultures in the economically important wheat crop, when crushed ME tissues were 2,4-D induced in culture. The macroscopic observation and the chronology of the morphologic events showed rapid cell proliferation, quickly followed by the formation of organized structures.

The regeneration process had occurred within a short time, from cell-cycle reactivation triggered by exogenously applied auxin treatment through to plantlet emergence. The appearance of chlorophyllous sectors was evidence of plastid differentiation, which occurs in parallel with the ongoing developmental differentiation process that takes place after synthetic auxin withdrawal.

### Wheat mature zygotic embryo competency: fundamental and practical issues

From earlier work (Chlyah et al. 1990; Eapen and Rao 1985) and our own previous results (Delporte et al. 2001, 2005), we showed in this study that the ME fragmentation produces good explants for tissue culture.

According to Chlyah et al. (1990) and Eapen and Rao (1985), embryo segmentation into two, four, or six pieces prevented the zygotic embryo from germinating and improved the morphogenesis rate via somatic embryogenesis. In two separate experiments, we observed high reproducibility in plant regeneration from MEs reduced to fragments (i.e., 22 % on average in terms of regenerated plants/ME; in Delporte et al. [2005] and in this study, for Minaret, the least responsive cultivar we have tested).

Since these reports, several studies have confirmed the potential of cutting the embryo into several pieces. Slightly damaging the radicle and plumule portions of MEs hampered root and bud formation and allowed higher quality and better callusing, whereas germination of MEs strongly reduced callus formation (Bi et al. 2007). More recently, using a series of conditions and components of the inductive medium tested, the ME scraping treatment proved to be the key step for an efficient regeneration system for this explant (broken into pieces instead of using the whole explant) (Yin et al. 2011).

Stress alone is known for promoting tissue culture response in several models (e.g., heavy metal, starvation, wounding) (Elhiti and Stasolla 2011). Biotic and abiotic stresses are implicit in dedifferentiation, with cells first acquiring a stem cell-like state and then assuming a new fate or dying if the stress level exceeds cellular tolerance or if the physiological state of the cells (depending on the genotype, cell identity, developmental state) does not allow a mediation of cellular stress responses (Fehér 2006, 2008; Grafi et al. 2011).

A close connection, or overlapping, between in vivo and in vitro plant morphogenesis and stress response pathways has frequently been highlighted (Gong et al. 2005; Tyburski and Tretyn 2010). The stress-induced morphogenic response is thought to be part of a general acclimation strategy, whereby plant growth is redirected to reduce stress exposure (Potters et al. 2007). Stress-related genome reprogramming has been experimentally observed during tissue culture response from wheat leaf base segments or ME explants, with the implication of genes in defense and anti-oxidation being observed during the expression of the developmental pathway in vitro (Delporte et al. 2013; Singla et al. 2007).

From a practical point of view, crushing MEs into thin pieces (500 μm in diameter) through a sterile nylon mesh and collecting them in a liquid medium before plating on a solid inductive medium is rather laborious and can cause contamination, whereas the scraping technique described by Yin et al. (2011) is easier to implement. Combined with an optimized culture medium, this physical pretreatment yielded 5–8 times more regeneration frequencies (compared with their basic protocol) and is therefore a useful alternative (Yin et al. 2011).

### Genotypic variation of morphogenetic events

It is necessary to screen wheat cultivars with good tissue culture response in order to accelerate the process of commercial field applications. Similarly, it is important to optimize a plant regeneration system with cultivars appropriate for breeding programs and commercial product development.

In this study, the competence of randomly selected commercial cultivars was demonstrated, and their morphogenetic capacities were compared when our regeneration model was applied. All agronomically valuable genotypes tested in this experiment produced plants, with variable efficiencies differently expressed at different developmental stages.

Among the several hundred fragments collected by grinding MEs (~500 fragments/~100 embryos), a great proportion of tissue pieces proliferated (90 %), and hence, the number of calli induced by initial explant (embryo) was much greater than that described in any other current callus-mediated method (i.e., 400–500 calli yielded from 100 embryos, corresponding to a 400–500 % callogenesis rate). The average values for morphogenesis was 60 % in the fourth week. In this study, given the initial number of calli that were induced, the number of morphogenic structures formed from a batch of 100 seeds after a 4-week induction period was very high (i.e., 200–300 morphogenic calli yielded, on average, from 100 embryos, corresponding to a 200–300 % embryogenesis rate). At this time, there was no significant difference among the genotypes tested.

Distinctive behavior among the varieties was evident in their ability to sustain the ongoing developmental process and/or continue to differentiate in the subsequent stages. After 7 weeks, the average proportion of morphogenic calli was still about 55 % across genotypes (i.e., at this time, the calli had a nodular appearance, almost all of them with green areas), but important differences were observed among varieties. The gap observed between the opposite values (73.80 and 21.98 %, for the most and least responsive varieties, respectively) reflected three scenarios: the proportion of calli being differentiated had dropped (Petrus and Minaret), had been maintained (Derwent), or had increased (Dream).

Later, differences emerged again, this time with regard to the efficiency of converting morphogenic structures into plantlet generation. High proportions of morphogenic calli generally produced higher regeneration rates. This was not true, however, for one variety (Derwent), which produced a high amount of morphogenic calli, similar to that produced by Dream, but had a lower (by 50 %) conversion rate of those calli into plants than Dream.

Plant regeneration from cultured tissues is genetically controlled, and a variable response is known across all genotypes and species (Bolibok and Rakoczy-Trojanowska 2006). The morphogenetic process is influenced by exogenous plant growth regulators and its interactions with endogenous phytohormones. The endogenous levels of storage products (protein or lipid, carbohydrate) in the explant are also known to be a determinant (de Almeida et al. 2012; Elmeer 2013; Gliwicka et al. 2012; Yaseen et al. 2012; Phillips 2004).

The frequency of in vitro plant regeneration of cereals is greatly influenced by genotype, and for ME culture, positive (but also negative) heterosis for in vitro character has been observed (Ozbay and Özgen 2010). In a comparative study of tissue culture response from MEs and IEs, substantial differences were observed between varieties for all the evaluated tissue culture parameters, corroborating the important role played by genotype throughout the regeneration process (Murín et al. 2012).

Genotype-dependent efficiency and the lack of correlation between total callus induction and regeneration from wheat MEs were reported by Zale et al. (2004). Other examples of the absence of a relationship between the frequency of callus induction and the quality of calli after a 2-week induction have been reported, but a high correlation has been recorded between callus differentiation frequency after a 6-week culture period and plantlet regeneration frequency (Bi et al. 2007). These authors suggested that callus differentiation was a key step in the tissue culture of *Triticum* MEs. In contrast, in their work on wheat ME tissue culture, Filippov et al. (2006) observed no significant relationship between the frequency of embryogenic callus formation and shoot regeneration (Filippov et al. 2006). A QTL mapping study for tissue culture performance indicated that the tissue culture response of different explant types of wheat could be controlled by shared or tightly linked genes, whereas different genes or gene combinations might govern the stages from callus induction to plantlet regeneration (Jia et al. 2007). Jia’s study and other reports have highlighted the importance of group 2 and 5 chromosomes in controlling tissue culture response in Triticeae crops.

In our study, different responses among varieties were recorded some weeks after the tissues had been transferred to regenerative conditions. This could indicate that some conditions, when applied as standards, might fail to meet the specific requirements of each genotype. As morphogenesis in vitro is a dynamic process, it is worth noting that not only might specific requirements be required at the initiation of the culture, but also that they might evolve differently over time.

Overall, the range of regeneration efficiency (defined as the number of plants regenerated/number of initial MEs) observed for our protocol when applied to commercial cultivars (15–35 %, an average of 22 %) is very similar to that (15–36.8 %) reported, for example, for Chinese elite cultivars (Yu et al. 2008). While for Yin et al. (2011), by applying the scraping technique and an improved culture medium composition, half of the 20 Chinese wheat lines out of 20 tested yielded a regeneration frequency of more than 30 % (2–85 %, an average of 28 %) (Yin et al. 2011)

Other studies intended to generate technical advances and create more robust regeneration protocols from IEs have opened up a new avenue for substantial improvements in ME-based regeneration models (Chauchan et al. 2007; Filippov et al. 2006). By manipulating various factors, such as more effective growth regulator combinations and time exposures adapted to the various genotypes, they have been able to greatly improve yields and reduce performance disparities between varieties. Another route would be the methodological option proposed by Eudes et al. (2003), which uses excised scutella from IEs as starting material but relies on inducing direct somatic embryogenesis, secondary embryogenesis, and regeneration without intermediate callus formation (Eudes et al. 2003).

### Ontogenesis and differentiation of somatic embryos

In order to understand the totipotency/pluripotency of somatic plant cells and to control in vitro regeneration, the genetic factors need to be identified, especially those involved in a specific morphogenic response induced in vitro. The molecular mechanisms induced after the hormone treatment of cultured cells to determine organogenesis or somatic embryogenesis are poorly understood, especially in monocot plants (Kraut et al. 2011; Su et al. 2007). A comparison of three transcriptomes in rice cell cultures (somatic embryogenesis, shoot organogenesis, and root organogenesis) pointed to a specific reorganization of the genome-wide transcriptional activities in relation to a particular morphogenic pathway induced in vitro (Su et al. 2007). A careful characterization of the plant regeneration system used as model is recommended in order to identify the genetic mechanisms that determine the organogenic or embryogenic response of cultured tissue.

Morphogenic and regenerable cell cultures are commonly described as “embryogenic.” Currently, embryogenic cultures remain the main route for cereal regeneration (Vasil 2005). The physical and morphological nature of so-called embryogenic callus cultures, which must by definition give rise to plants through the formation of somatic embryos, is not by itself an identifying criterion.

Because of the close visual resemblance between the external appearance of regenerating structures that can be organogenic and/or embryogenic, and because the morphology of the regenerated structures is not enough to classify them as initiated from true somatic embryos, it is necessary to prove histologically that regenerating structures develop via stages similar to those of zygotic embryos developing into individual structures with clear bipolarity (Nowak et al. 2012; Salaj et al. 2005).

This is particularly true of cereals, where induced embryos are often macroscopically similar to shoots or leaves, and the proper identification of regenerative pathways is therefore difficult (Konieczny et al. 2003). Totipotent calli are commonly described as dense nodular structures that are yellowish-white, later forming green spots and then green buds, followed by regeneration, but a careful examination of the cultures under the microscope has rarely been performed. By definition, somatic embryos are bipolar structures, and therefore, the presence of shoot and root apical meristems is recommended as the main marker of the true embryonic nature of the regenerated structure. Unfortunately, as histological analysis can be laborious and time consuming, the formation of two poles and the full development of the regenerated embryo structures have seldom been documented. In most of the embryoids, root development was delayed until after shoot formation, although a distinct polarity was evident at the time of shoot initiation (Fernandez et al. 1999; Ozias-Akins and Vasil 1983a).

The somatic embryogenesis pathway has been often described in *Triticum* sp. cultures, but organogenesis has also occurred, exclusively or concomitantly (Bartók and Sági 1990; Bennici and d’Amato 1978; Eapen and Rao 1985; Konieczny et al. 2003; Ozias-Akins and Vasil 1983a, b, 1982; Bennici et al. 1988; He et al. 1990; Kothar and Varshney 1998), and somatic embryogenesis alone has rarely been observed (Fernandez et al. 1999; Ozias-Akins and Vasil 1983a).

This paper has described a histological study of *T. aestivum* L. somatic embryo formation, from their first cellular divisions to the last step of their complete development, passing through a chronological series of characteristic morphological stages (initial cell division, cell proliferation, meristematic clumps, pro-embryo, globular embryo surrounded by the protoderm, polarized structure with meristematic cells forming a caulinar pole without any vascular connection with the original tissue, bipolarized and fully differentiated somatic embryo with a well-developed scutellum and vascular connections established between the shoot and root apical meristems, showing all the characteristic organs of grass embryos). The complete phase in these wheat cultures lasts about 3 weeks. This reflects an embryonic differentiation process that is faster than that observed in cultures of *Triticum durum* Desf. IEs, where fully developed somatic embryos with a scutellum-like structure were observed after 5 weeks (Fernandez et al. 1999).

Somatic embryos can differentiate either directly from the explant without an intervening callus phase or indirectly after a callus phase (Williams and Maheswaran 1986), but the distinction between the direct and indirect process is unclear (von Arnold et al. 2002). In our study, although concomitant callus development was demonstrated, given the short period over which the process was carried out, our results suggest that direct somatic embryogenesis occurred. This timing is similar to that described previously as a process of direct somatic embryogenesis in wheat (i.e., within 1 week, globular somatic embryos were formed) (Eudes et al. 2003).

In *Triticum* sp., while the compact, yellowish, nodular callus is derived from the epithelial and sub-epithelial cells of the IE scutellum (Fernandez et al. 1999; Ozias-Akins and Vasil 1982), the ME callus is thought to derive from tissues within and near the procambium of the embryo axis (Ozias-Akins and Vasil 1983b). After seed germination, mesocotyl tissue with the scutellum was used by Bartók and Sági (1990) for callus induction, but histological analysis showed the plantlets regenerated via organogenesis (Bartók and Sági 1990). Plants had also been regenerated from mesocotyl segments of germinated ME in *T. durum* (Bennici and d’Amato 1978). In addition, epidermal cells of leaf or coleoptile bases in barley (*Hordeum vulgare* L.) and shoot apices in finger millet (*Eleusine coracana* GAERTN) were thought to be possible origins of the regeneration pathway (Eapen and George 1990; Oka et al. 1995).

Small fragments were used in our study, but the cultivation of tissues obtained from embryos dissected into their main parts (detached or not from the scutellum, separating the three main anatomical regions of the embryo axis: root pole, mesocotyl, and shoot pole) allowed us to assess and localize the tissue origin of the regenerated plants.

### Cytological and physiological aspects

Reentry into the cell cycle plays a crucial role in the expression of cellular totipotency (Gutierrez et al. 2002). The initiation of cell proliferation, the first visible stage in any regeneration process, is rapidly and intensely stimulated in the presence of the 2,4-D growth regulator (Fig. 3a–c). Cell division induction arises preferentially in the embryo tissue corresponding to the mesocotyl and in the presence of scutellum tissue. Thereafter, some of the dividing cells proceed towards morphogenesis (Fig. 3d–g). The presence of the scutellum could be a positive factor triggering the sequence of the early processes as a nutrient supplier to the responsive cells.

First, the activation of cell division occurs earlier in cultures of MEs than those of IEs (Fernandez et al. 1999; Ortiz et al. 1996; Ozias-Akins and Vasil 1982), probably because this physiological step has already been activated during imbibition of the caryopses, the first phase of explant processing in our methodology. The physiological responses are the result of the integration of many events transduced into a comprehensive network of signaling pathways, and plant hormones occupy a central place in this network (Wu et al. 2007). The hormonal signals that occur upon dormancy release create a first wave of chemical stimuli in the quiescent zygotic embryo. These stimuli are able to induce germination (i.e., the metabolic network that fuels the initial burst of cell proliferation and resumption of growth of the embryo towards the seedling) (Bethke et al. 1997; Bewley 1997; del Pozo et al. 2005; Kucera et al. 2005; Olszewski et al. 2002). In addition, during somatic embryogenesis induction, the acquisition of embryogenic competence also goes through a proliferation stage (Namasivayam 2007); in our protocol, this stage might have been primed during seed imbibition, leading to the activation of metabolic processes from an initial quiescent state.

Second, the initiation of this mitotic process is located in the mesocotyl region, whereas in the cultures of cereal IEs (Magnusson and Bornmann 1985; Ozias-Akins and Vasil 1982), including those of wheat (Fernandez et al. 1999; Heyser et al. 1984), embryogenic cultures originate through the proliferation of the epithelial and sub-epithelial layers of the scutellum. This dissimilarity might be explained by the different physiological states of the zygotic ME and its immature counterpart. In our protocol, the specific receptiveness of mesocotyl tissue to the in vitro culture conditions could be ascribed to differing sensitivity (compared with the scutellum) to the auxin-dependent reactivation of cell cycle progression. During seed maturation, cells from embryonic tissues stop dividing at different phases of the cell cycle. Baíza and Sànchez-de-Jiménez (1989) observed cell division reactivation in maize seeds during both germination and auxin-induced in vitro culture; from the different tissues constituting the embryonic axes, the mesocotyl was the first to divide (Baíza and Sànchez-de-Jiménez 1989). Therefore, the mesocotyl cells treated according to our protocol would require short 2,4-D exposure to rapidly commit to the embryogenic pathway. Those cells requiring minimal additional stimuli to become embryogenic are defined as competent (Toonen et al. 1994).

Third, the positive influence of the scutellum might be due to positional effects of “nutrient-rich” neighboring cells. During cereal germination, the scutellum is a specialized organ in the mobilization and transfer of nutrients from the storage tissue to the zygotic embryo. Organogenesis is an energy-consuming process. Starch accumulation/mobilization cycles appear to be a characteristic feature of cells involved in plant morphogenesis, including somatic embryogenesis (Fortes and Pais 2000; Ho and Vasil 1983; Martin et al. 2000), and the physical influence of neighboring cells has been shown to be a valuable contributor.

## Conclusion

The regeneration of plants from cultured cells and tissues of wheat, like other grasses and monocotyledonous species, has long been thought to be very difficult, if not impossible (Vasil 2007).

Obviously, the embryogenic ability of plant cells continuously decreases during plant ontogenesis, and it is species-dependent. In monocotyledonous plants, including most of the agronomically important cereals, embryogenic competence is restricted mainly to cells of embryogenic or meristematic origin, including IEs or seeds and leaf bases (Fehér 2006).

Highly efficient and reproducible regeneration methods are needed for large-scale applications in plant biotechnology. Such high-frequency regeneration protocols have been established for rice, based on the culture of mature seeds. Although important advances have been made in the field of Triticeae, a highly efficient and cost-effective regeneration system (similar to those available for rice) using wheat MEs is still not available for all genotypes. Extensive work is currently being conducted in order to improve the understanding and control of the expression of totipotency in wheat, notably in recent years by using zygotic MEs as explants (Jia et al. 2007; Tang et al. 2006; Yin et al. 2011; Yu et al. 2008; Coskun et al. 2013; Miroshnichenko et al. 2013; Murín et al. 2012; Ozbay and Özgen 2010; Parmar et al. 2012).

In this study, although ME fragments appear promising for developing an efficient and reproducible regeneration protocol, there is a striking discrepancy between (1) the high callusing rate and high proportion of calli involved in the differentiation stage and (2) their ultimate involvement in the regeneration stage, which is lower. A better understanding of the specific requirements of these explants at each critical step of the regeneration process is needed in order to improve their entire regenerative performance.

Dynamic changes in cellular physiology and metabolism, dedifferentiation, revival/renewal of cell division, reprogramming and acquisition of new developmental fate, and morphogenesis in response to hormonal stimuli all represent processes initiated by profound molecular changes. The ability of cultured explant tissue to reset its epigenetic regulation of gene expression in order to withstand the artificial and sub-optimal environment is subject to multiple influences. Factors influencing in vitro adaptability and regeneration are varied, ranging from genotype and origin and type of explant to hormonal effects. Because of genotypic specificity, media and the cultural environment often need to be varied from one genus or species of plant to another (Elmeer 2013; Neelakandan and Wang 2012).

As noted by Schulze (2007), after “more than fifty years of cereal tissue culture, extensive and detailed studies … revealed various factors dramatically influencing morphogenic competence. A lot of data accumulated on the interaction of these parameters suggested that the development and use of genotype specific protocols by systematic testing of a range of key variables can enhance plant regeneration” (Schulze 2007).

By using mature caryopses ground into fairly thin pieces as starting material, our method has the advantage of producing a significant quantity of small calli, and a large number of cells are therefore physically accessible to direct DNA delivery, compared with the bigger cell masses proliferating from the culture of entire embryos

For a long time, IEs were the preferred explants for initiating wheat regenerable cultures. The poorer performance of MEs usually evaluated with protocols optimized for immature explants could have led to the persistent underestimation of their potential. The plant regeneration mechanism in the two in vitro regeneration systems (ME vs. IE culture) might be different enough, however, to hamper the development of an optimal plant regeneration protocol for use in both systems (Murín et al. 2012). Recent studies, by demonstrating comparable or even higher ME performances, highlight the need to improve protocols by taking account of the genotype and explant type specificities. Very recent studies show encouraging results in this regard by further optimizing the overall determinant factors and improving the in vitro culture response from MEs (Battal 2010; Ding et al. 2009; Miroshnichenko et al. 2009; Moghaieb et al. 2010; Murín et al. 2012; Ozbay and Özgen 2010; Parmar et al. 2012; Rashid et al. 2009; Ren et al. 2010; Yin et al. 2011).

## Abbreviations

2,4-D: 2,4-dichlorophenoxyacetic acid
ME: Mature embryo
IE: Immature embryo

## References

[CR1] Arias RS, Filichkin SA, Strauss SH (2006). Divide and conquer: development and cell cycle genes in plant transformation. Trends Biotechnol.

[CR2] Baíza AM, Sànchez-de-Jiménez E (1989). Effect of the auxin, 2-(2-methyl-4-chloro-phenoxy)propionic acid, on cell cycle regulation in maize embryonic tissues. Physiol Plant.

[CR3] Bartók T, Sági F (1990). A new, endosperm-supported callus induction method for wheat (Triticum aestivum L.). Plant Cell Tissue Organ Cult.

[CR4] Battal A (2010) Optimization of mature embryo based regeneration and genetic transformation of Turkish wheat cultivars. Thesis. Graduate School of Natural and Applied Sciences, Middle East Technical University

[CR5] Bennici A, d’Amato F (1978). In vitro regeneration of durum wheat plants, 1. Zeitschrift fuer Pflanzenzuechtung.

[CR6] Bennici A, Caffaro L, Dameri R, Gastaldo P, Profumo P (1988). Callus formation and plantlet regeneration from immature Triticum durum Desf. embryos. Euphytica.

[CR7] Bethke PC, Schuurink R, Jones RL (1997). Hormonal signalling in cereal aleurone. J Exp Bot.

[CR8] Bewley JD (1997). Seed germination and dormancy. Plant Cell.

[CR9] Bhaskaran S, Smith RH (1990). Regeneration in cereal tissue culture: a review. Crop Sci.

[CR10] Bhojwani SS, Razdan MK (1986) Plant tissue culture: theory and practice. Access Online via Elsevier

[CR11] Bi RM, Kou M, Chen LG, Mao SR, Wang HG (2007). Plant regeneration through callus initiation from mature embryo of Triticum. Plant Breed.

[CR12] Bolibok H, Rakoczy-Trojanowska M (2006). Genetic mapping of QTLs for tissue-culture response in plants. Euphytica.

[CR13] Chauchan H, Desai SA, Khurana P (2007). Comparative analysis of the differential regeneration response of various genotypes of Triticum aestivum, Triticum durum and Triticum dicoccum. Plant Cell Tissue Organ Cult.

[CR14] Chlyah H, Hsaine M, Karim R, Chlyah A, Bajaj YPS (1990). Improvement of somatic embryogenesis in wheat by segmentation of cultured embryos. Wheat.

[CR15] Coskun Y, Duran RE, Savaskan C, Demirci T, Hakan MT (2013). Efficient plant regeneration with arabinogalactan-proteins on various ploidy levels of cereals. J Integr Agric.

[CR16] de Almeida M, de Almeida CV, Graner EM, Brondani GE, de Abreu-Tarazi MF (2012). Pre-procambial cells are niches for pluripotent and totipotent stem-like cells for organogenesis and somatic embryogenesis in the peach palm: a histological study. Plant Cell Rep.

[CR17] del Pozo JC, Lopez-Matas MA, Ramirez-Parra E, Gutierrez C (2005). Hormonal control of the plant cell cycle. Physiol Plant.

[CR18] Delporte F, Mostade O, Jacquemin JM (2001). Plant regeneration through callus initiation from thin mature embryo fragments of wheat. Plant Cell Tissue Organ Cult.

[CR19] Delporte F, Li S, Jacquemin JM (2005). Calluses initiated from thin mature embryo fragments are suitable targets for wheat transformation as assessed by long-term GUS expression studies. Plant Cell Tissue Organ Cult.

[CR20] Delporte F, Muhovski Y, Pretová A, Watillon B (2013). Analysis of expression profiles of selected genes associated with the regenerative property and the receptivity to gene transfer during somatic embryogenesis in *Triticum aestivum* L. Mol Biol Rep.

[CR21] Ding L, Li S, Gao J, Wang Y, Yang G, He G (2009). Optimization of Agrobacterium-mediated transformation conditions in mature embryos of elite wheat. Mol Biol Rep.

[CR22] Eapen S, George L (1990). Influence of phytohormones, carbohydrates, aminoacids, growth supplements and antibiotics on somatic embryogenesis and plant differentiation in finger millet. Plant Cell Tissue Organ Cult.

[CR23] Eapen S, Rao P (1985). Factors controlling callus initiation, growth and plant regeneration in breadwheat (Triticum aestivum L). Proc Plant Sci.

[CR24] Elhiti M, Stasolla C (2011) The use of zygotic embryos as explants for in vitro propagation: an overview. In: Thorpe TA, Yeung EC (eds) Plant embryo culture, vol 710. Methods in molecular biology. Humana Press, pp 229-255. doi:10.1007/978-1-61737-988-8_17 10.1007/978-1-61737-988-8_17 21207273

[CR25] Elmeer KES (2013) Factors regulating somatic embryogenesis in plants. In: Aslam J, Srivastava PS, Sharma MP (eds) Somatic Embryogenesis and Gene Expression. Narosa Publishing House, New Delhi, pp 56-81.

[CR26] Eudes F, Acharya S, Laroche A, Selinger LB, Cheng K-J (2003). A novel method to induce direct somatic embryogenesis, secondary embryogenesis and regeneration of fertile green cereal plants. Plant Cell Tissue Organ Cult.

[CR27] Fehér A, Mujib A, Šamaj J (2006). Why somatic plant cells Start to form embryos?. Somatic embryogenesis.

[CR28] Fehér A (2008). The initiation phase of somatic embryogenesis: what we know and what we don't. Acta Biologica Szegediensis.

[CR29] Fernandez S, Michaux-Ferriere N, Coumans M (1999). The embryogenic response of immature embryo cultures of durum wheat (Triticum durum Desf.): Histology and improvement by AgNO3. Plant Growth Regul.

[CR30] Filippov M, Miroshnichenko D, Vernikovskaya D, Dolgov S (2006). The effect of auxins, time exposure to auxin and genotypes on somatic embryogenesis from mature embryos of wheat. Plant Cell Tissue Organ Cult.

[CR31] Finer JJ (2010). Plant nuclear transformation.

[CR32] Fortes AM, Pais MS (2000). Organogenesis from internode-derived nodules of Humulus lupulus var. Nugget (Cannabinaceae): histological studies and changes in the starch content. Am J Bot.

[CR33] Gliwicka M, Nowak K, Cieśla E, Gaj M (2012). Expression of seed storage product genes (CRA1 and OLEO4) in embryogenic cultures of somatic tissues of Arabidopsis. Plant Cell Tissue Organ Cult.

[CR34] Gong H, Jiao Y, Hu W-W, Pua E-C (2005). Expression of glutathione-S-transferase and its role in plant growth and development in vivo and shoot morphogenesis in vitro. Plant Mol Biol.

[CR35] Grafi G, Chalifa-Caspi V, Nagar T, Plaschkes I, Barak S, Ransbotyn V (2011). Plant response to stress meets dedifferentiation.

[CR36] Gutierrez C, Ramirez-Parra E, Castellano MM, Del Pozo JC (2002). G(1) to S transition: more than a cell cycle engine switch. Curr Opin Plant Biol.

[CR37] He D, Yang Y, Bertram J, Scott K (1990). The histological development of the regenerative tissue derived from cultured immature embryos of wheat (*Triticum aestivum* L.). Plant Sci.

[CR38] Heyser JW, Nabors MW, MacKinnon C, Dykes TA, Demott KJ, Kautzman DC, Mujeeb-Kazi A (1984). Long-term, high-frequency plant regeneration and the induction of somatic embryogenesis in callus cultures of wheat (Triticum aestivum L.). Z Pflanzenzüchtung.

[CR39] Hicks G (1994). Shoot induction and organogenesis in vitro: a developmental perspective. Vitro Cell Dev Biol Plant.

[CR40] Ho WJ, Vasil IK (1983). Somatic embryogenesis in sugar cane (Saccharum officinarum L.): growth and plant regeneration from embryogenic cell suspension cultures. Ann Bot.

[CR41] Jeong D-H, An S, Kang H-G, Moon S, Han J-J, Park S, Lee HS, An K, An G (2002). T-DNA insertional mutagenesis for activation tagging in rice. Plant Physiol.

[CR42] Jia H, Yi D, Yu J, Xue S, Zhang C, Zhang Z, Zhang L, Ma Z, Xiang Y (2007). Mapping QTLs for tissue culture response of mature wheat embryos. Mol Cells.

[CR43] Jia H, Yu J, Yi D, Cheng Y, Xu W, Zhang L, Ma Z (2009). Chromosomal intervals responsible for tissue culture response of wheat immature embryos. Plant Cell Tissue Organ Cult.

[CR44] Jiménez VM (2001). Regulation of in vitro somatic embryogenesis with emphasis on to the role of endogenous hormones. Rev Bras Fisiol Veg.

[CR45] Konieczny R, Czaplicki A, Golczyk H, Przywara L (2003). Two pathways of plant regeneration in wheat anther culture. Plant Cell Tissue Organ Cult.

[CR46] Kothar S, Varshney A (1998). Morphogenesis in long-term maintained immature embryo-derived callus of wheat (Triticum aestivum L)—histological evidence for both somatic embryogenesis and organogenesis. J Plant Biochem Biotechnol.

[CR47] Kraut M, Wójcikowska B, Ledwoń A, Gaj MD (2011). Immature zygotic embryo cultures of Arabidopsis. A model system for molecular studies on morphogenic pathways induced In vitro. Acta Biol Cracov Ser Bot.

[CR48] Kucera B, Cohn MA, Leubner-Metzger G (2005). Plant hormone interactions during seed dormancy release and germination. Seed Sci Res.

[CR49] Lee S, Jeon J-S, Jung K-H, An G (1999). Binary vectors for efficient transformation of rice. J Plant Biol.

[CR50] Magnusson I, Bornmann CH (1985). Anatomical observations on somatic embryogenesis from scutellar tissue of immature zygotic embryos of Triticum aestivum. Physiol Plant.

[CR51] Martin AB, Cuadrado Y, Guerra H, Gallego P, Hita O, Martin L, Dorado A, Villalobos N (2000). Differences in the contents of total sugars, reducing sugars, starch and sucrose in embryogenic and non-embryogenic calli from Medicago arborea L. Plant Sci.

[CR52] Miroshnichenko D, Filippov M, Dolgov S (2009). Effects of daminozide on somatic embryogenesis from immature and mature embryos of wheat. AJCS.

[CR53] Miroshnichenko D, Poroshin G, Dolgov S (2011). Genetic transformation of wheat using mature seed tissues. Appl Biochem Microbiol.

[CR54] Miroshnichenko DN, Filippov MV, Dolgov SV (2013). Medium optimization for efficient somatic embryogenesis and in vitro plant regeneration of spring common wheat varieties. Russ Agric Sci.

[CR55] Moghaieb RE, El-Arabi NI, Momtaz OA, Youssef SS, Soliman MH (2010). Genetic transformation of mature embryos of bread (T. aestivum) and pasta (T. durum) wheat genotypes. GM Crops.

[CR56] Murín R, Mészáros K, Nemeček P, Kuna R, Faragó J (2012). Regeneration of immature and mature embryos from diverse sets of wheat genotypes using media containing different auxins. Acta Agron Hung.

[CR57] Nakamura H, Hakata M, Amano K, Miyao A, Toki N, Kajikawa M, Pang J, Higashi N, Ando S, Toki S, Fujita M, Enju A, Seki M, Nakazawa M, Ichikawa T, Shinozaki K, Matsui M, Nagamura Y, Hirochika H, Ichikawa H (2007). A genome-wide gain-of-function analysis of rice genes using the FOX-hunting system.

[CR58] Namasivayam P (2007). Acquisition of embryogenic competence during somatic embryogenesis. Plant Cell Tissue Organ Cult.

[CR59] Neelakandan A, Wang K (2012). Recent progress in the understanding of tissue culture-induced genome level changes in plants and potential applications. Plant Cell Rep.

[CR60] Nowak K, Wojcikowska B, Szyrajew K, Gaj MD (2012). Evaluation of different embryogenic systems for production of true somatic embryos in Arabidopsis. Biol Plant.

[CR61] Oka S, Saito N, Kawaguchi H (1995). Histological observations on initiation and morphogenesis in immature and mature embryo derived callus of barley (Hordeum vulgare L.). Ann Bot.

[CR62] Olszewski N, T-p S, Gubler F (2002). Gibberellin signaling: biosynthesis, catabolism, and response pathways. Plant Cell.

[CR63] Ortiz JPA, Fama G, Vallejos RH, De Halac IN (1996). Cytodifferentiation and cell organization in the somatic embryogenesis of wheat (Triticum aestivum L). Biocell.

[CR64] Ozbay A, Özgen M (2010). Is heterosis noticeable in the callus response of winter durum wheat F1 hybrids?. Biol Plant.

[CR65] Ozias-Akins P, Vasil IK (1982). Plant regeneration from cultured immature embryos and inflorescences of Triticum aestivum L. (wheat): evidence for somatic embryogenesis. Protoplasma.

[CR66] Ozias-Akins P, Vasil I (1983). Improved efficiency and normalization of somatic embryogenesis in *Triticum aestivum* (wheat). Protoplasma.

[CR67] Ozias-Akins P, Vasil IK (1983). Callus induction and growth from the mature embryo of Triticum aestivum L. (wheat). Protoplasma.

[CR68] Parmar S, Sainger M, Chaudhary D, Jaiwal P (2012). Plant regeneration from mature embryo of commercial Indian bread wheat (Triticum aestivum L.) cultivars. Physiol Mol Biol Plants.

[CR69] Phillips GC (2004). In vitro morphogenesis in plants-recent advances. Vitro Cell Dev Biol Plant.

[CR70] Potters G, Pasternak TP, Guisez Y, Palme KJ, Jansen MAK (2007). Stress-induced morphogenic responses: growing out of trouble?. Trends Plant Sci.

[CR71] Rashid U, Ali S, Ali GM, Ayub N, Masood MS (2009). Establishment of an efficient callus induction and plant regeneration system in Pakistani wheat (Triticum aestivum) cultivars. Electron J Biotechnol.

[CR72] Ren J-P, Wang X-G, Yin J (2010). Dicamba and sugar effects on callus induction and plant regeneration from mature embryo culture of wheat. Agric Sci China.

[CR73] Saika H, Toki S (2009). Towards a highly efficient gene targeting system in higher plants. JARQ.

[CR74] Saika H, Toki S (2010). Mature seed-derived callus of the model *indica* rice variety Kasalath is highly competent in *Agrobacterium*-mediated transformation. Plant Cell Rep.

[CR75] Saika H, Oikawa A, Matsuda F, Onodera H, Saito K, Toki S (2011). Application of gene targeting to designed mutation breeding of high-tryptophan rice. Plant Physiol.

[CR76] Salaj J, Petrovská B, Obert B, Pret'ová A (2005). Histological study of embryo-like structures initiated from hypocotyl segments of flax (Linum usitatissimum L.). Plant Cell Rep.

[CR77] Sallaud C, Gay C, Larmande P, Bès M, Piffanelli P, Piégu B, Droc G, Regad F, Bourgeois E, Meynard D, Périn C, Sabau X, Ghesquière A, Glaszmann JC, Delseny M, Guiderdoni E (2004). High throughput T-DNA insertion mutagenesis in rice: a first step towards in silico reverse genetics. Plant J.

[CR78] Schulze J (2007). Improvements in cereal tissue culture by thidiazuron: a review. Fruit Veg Cereal Sci Biotechnol.

[CR79] Shewry PR, Jones HD (2005). Transgenic wheat: where do we stand after the first 12 years?. Ann Appl Biol.

[CR80] Shrawat AK, Good AG (2010). A high-throughput Agrobacterium tumefaciens-mediated transformation system for molecular breeding and functional genomics of rice (*Oryza sativa* L.). Plant Biotechnol.

[CR81] Singla B, Tyagi AK, Khurana JP, Khurana P (2007). Analysis of expression profile of selected genes expressed during auxin-induced somatic embryogenesis in leaf base system of wheat (*Triticum aestivum*) and their possible interactions. Plant Mol Biol.

[CR82] Su N, He K, Jiao Y, Chen C, Zhou J, Li L, Bai S, Li X, Deng XW (2007). Distinct reorganization of the genome transcription associates with organogenesis of somatic embryo, shoots, and roots in rice. Plant Mol Biol.

[CR83] Tahir M, Waraich E, Stasolla C (2011) Genetic transformation protocols using zygotic embryos as explants: an overview. In: Thorpe TA, Yeung EC (eds) Plant embryo culture, vol 710. Methods in molecular biology. Humana Press, pp 309-326. doi:10.1007/978-1-61737-988-8_21 10.1007/978-1-61737-988-8_21 21207277

[CR84] Tang Z-X, Ren Z-L, Wu F, Fu S-L, Wang X-X, Zhang H-Q (2006). The selection of transgenic recipients from new elite wheat cultivars and study on its plant regeneration system. Agric Sci China.

[CR85] Terada R, Asao H, Iida S (2004). A large-scale *Agrobacterium*-mediated transformation procedure with a strong positive-negative selection for gene targeting in rice (*Oryza sativa* L.). Plant Cell Rep.

[CR86] Toki S, Hara N, Ono K, Onodera H, Tagiri A, Oka S, Tanaka H (2006). Early infection of scutellum tissue with Agrobacterium allows high-speed transformation of rice. Plant J.

[CR87] Toonen MAJ, Hendriks T, Schmidt EDL, Verhoeven HA, van Kammen A, deVries SC (1994). Description of somatic-embryo forming single cells in carrot suspension cultures employing video cell tracking. Planta.

[CR88] Tyburski J, Tretyn A, Nasar A, Chan M-T, Umar S (2010). Ascorbate and glutathione in organogenesis, regeneration and differentiation in plant in vitro cultures. Ascorbate-glutathione pathway and stress tolerance in plants.

[CR89] Vasil IK (1988). Progress in the regeneration and genetic manipulation of cereal crops. Biotechnology.

[CR90] Vasil IK (2005). The story of transgenic cereals: the challenge, the debate, and the solution—a historical perspective. In Vitro Cell Dev Biol Plant.

[CR91] Vasil I (2007). Molecular genetic improvement of cereals: transgenic wheat (Triticum aestivum L.). Plant Cell Rep.

[CR92] Vasil IK (2008). A history of plant biotechnology: from the cell theory of Schleiden and Schwann to biotech crops. Plant Cell Rep.

[CR93] von Arnold S, Sabala I, Bozhkov P, Dyachok J, Filonova L (2002). Developmental pathways of somatic embryogenesis. Plant Cell Tissue Organ Cult.

[CR94] Williams EG, Maheswaran G (1986). Somatic embryogenesis: factors influencing coordinated behaviour of cells as an embryogenic group. Ann Bot.

[CR95] Wu G, Shao HB, Chu LY, Cai JW (2007). Insights into molecular mechanisms of mutual effect between plants and the environment. A review. Agron Sustain Dev.

[CR96] Xia L, Ma Y, He Y, Jones HD (2012). GM wheat development in China: current status and challenges to commercialization. J Exp Bot.

[CR97] Yang Y, Peng H, Huang H, Wu J, Jia S, Huang D, Lu T (2004). Large-scale production of enhancer trapping lines for rice functional genomics. Plant Sci.

[CR98] Yang Y, Li Y, Wu C (2013). Genomic resources for functional analyses of the rice genome. Curr Opin Plant Biol.

[CR99] Yaseen M, Ahmad T, Sablok G, Standardi A, Hafiz IA (2013) Review: role of carbon sources for in vitro plant growth and development. Mol Biol Rep 40(4):2837-2849 10.1007/s11033-012-2299-z 23212616

[CR100] Yin G-X, Wang Y-L, She M-Y, Du L-P, Xu H-J, Ma J-X, Ye X-G (2011). Establishment of a highly efficient regeneration system for the mature embryo culture of wheat. Agric Sci China.

[CR101] Yu Y, Wang J, Zhu ML, Wei ZM (2008). Optimization of mature embryo–based high frequency callus induction and plant regeneration from elite wheat cultivars grown in China. Plant Breed.

[CR102] Zale JM, Borchardt-Wier H, Kidwell KK, Steber CM (2004). Callus induction and plant regeneration from mature embryos of a diverse set of wheat genotypes. Plant Cell Tissue Organ Cult.

